# O papel mediador da dependência de mídia social e da qualidade do sono na associação entre tempo de uso de mídia social e sintomas depressivos em universitários

**DOI:** 10.1590/0102-311XPT097423

**Published:** 2024-06-14

**Authors:** Jéssica Vertuan Rufino, Renne Rodrigues, Arthur Eumann Mesas, Camilo Molino Guidoni

**Affiliations:** 1 Universidade Estadual de Londrina, Londrina, Brasil.; 2 Universidade Federal da Fronteira Sul, Chapecó, Brasil.; 3 Health and Social Research Center, Universidad de Castilla-La Mancha, Cuenca, España.

**Keywords:** Mídias Sociais, Sintomas Depressivos, Qualidade do Sono, Adulto Jovem, Análise de Mediação, Social Media, Depressive Symptoms, Sleep Quality, Young Adult, Mediation Analysis, Medios de Comunicación Sociales, Síntomas Depresivos, Calidad del Sueño, Adulto Joven, Análisis de Mediación

## Abstract

O aumento do uso de mídias sociais e sua associação com sintomas depressivos, especialmente em jovens adultos, tem gerado a necessidade do entendimento de como ocorre tal associação para subsidiar políticas de redução de danos e agravos. Nesse sentido, este estudo objetivou verificar o efeito mediador da dependência de mídias sociais e da qualidade do sono na associação entre o tempo de uso de mídias sociais e sintomas depressivos em universitários brasileiros. Trata-se de um estudo transversal, realizado com 2.823 universitários, que forneceram informações referentes ao tempo de uso de mídias sociais, à dependência de mídias sociais, aos sintomas depressivos e à qualidade do sono. A análise de mediação, ajustada por fatores de confusão, foi realizada por meio do software PROCESS para SPSS, para obtenção do efeito total (c), direto (c’) e indiretos (EI_1_, EI_2_ e EI_3_). Os resultados identificaram associação entre o tempo de uso de mídias sociais e os sintomas depressivos, mediada pela dependência de mídias sociais (EI_1_ = 20%) e pela qualidade do sono (EI_1_ = 40%). Os resultados permitem ampliar o conhecimento acerca dos mecanismos que influenciam mutuamente a relação entre o tempo de uso de mídias sociais e os sintomas depressivos, auxiliando na adoção de estratégias de redução de danos decorrentes do uso excessivo de mídias sociais.

## Abstract (en)

Increase in time spent on social media and its association with depressive symptoms, especially among young adults, has generated the need to understand how this association occurs in order to support the development of policies to reduce harm and complications. In view of this fact, this study aimed to assess the mediating effect of social media addiction and sleep quality on the association between time spent on social media and depressive symptoms in Brazilian university students. This is a cross-sectional study conducted with 2,823 university students, who provided information regarding time spent on social media, social media addiction, depressive symptoms and sleep quality. The mediation analysis, adjusted for confounding factors, was performed using the PROCESS macro for SPSS to obtain the total (c), direct (c’), and indirect effects (EI_1_, EI_2_, and EI_3_). The results identified an association between time spent on social media and depressive symptoms, mediated by social media addiction (EI_1_ = 20%) and sleep quality (EI_1_ = 40%). These findings help expand knowledge about the mechanisms that mutually influence the relationship between time spent on social media and depressive symptoms, supporting the adoption of strategies to reduce harm resulting from excessive social media use.

## Resumen

El aumento del uso de las redes sociales y su asociación con síntomas depresivos, especialmente en adultos jóvenes, ha generado la necesidad de comprender cómo se produce esta asociación como una forma de apoyar las políticas de reducción de daños y afecciones. En ese sentido, el presente estudio tuvo como objetivo verificar el efecto mediador de la adicción a las redes sociales y de la calidad del sueño en la asociación entre el tiempo de uso de las redes sociales y los síntomas depresivos en estudiantes universitarios brasileños. Se trata de un estudio transversal, realizado con 2.823 estudiantes universitarios, quienes brindaron información respecto al tiempo de uso de las redes sociales, adicción a las redes sociales, síntomas depresivos y calidad del sueño. El análisis de mediación, ajustado por factores de confusión, se realizó por medio del software PROCESS para SPSS, para obtener el efecto total (c), directo (c’) e indirectos (EI_1_, EI_2_ y EI_3_). Los resultados identificaron una asociación entre el tiempo de uso de las redes sociales y los síntomas depresivos, mediada por adicción a las redes sociales (EI_1_ = 20%) y la calidad del sueño (EI_1_ = 40%). Los resultados permiten ampliar el conocimiento acerca de los mecanismos que influyen mutuamente en la relación entre tiempo de uso de las redes sociales y los síntomas depresivos, ayudando a adoptar estrategias para reducir los daños resultantes del uso excesivo de las redes sociales.

## Introdução

O uso da internet e suas ferramentas permite um acesso sem precedentes à informação, possibilitando meios para que 64,4% da população mundial, no início de 2023 ^1^, possa se comunicar, trabalhar e/ou se entreter de forma *online* ^2^ ^,^ ^3^. Embora sejam possibilitadas diversas ações ^3^ (*streaming* de vídeos, leitura, músicas, jogos etc.), o uso de mídias sociais (WhatsApp, Facebook, Instagram, TikTok, entre outros) apresentou um crescimento exponencial desde 2005, tanto em países desenvolvidos como em desenvolvimento ^4^ ^,^ ^5^, sendo acessadas por 59,4% da população mundial em 2023, com maior prevalência na Nigéria e no Brasil ^1^. Os jovens adultos constituem o estrato etário com maior frequência (variando de 53,4 a 96% em diferentes países) ^3^ e maior tempo ^1^ ^,^ ^3^ ^,^ ^6^ (média diária de 3h11min para mulheres e 2h46min para homens) ^1^ de uso de mídias sociais.

Apesar das vantagens propiciadas pelas mídias sociais, o seu uso crescente é associado à diminuição do bem-estar ^4^, à piora da qualidade do sono ^7^ e a problemas relacionados à saúde mental ^2^ ^,^ ^8^, como a depressão ^2^. Entre os principais sintomas clínicos da depressão, destacam-se: humor deprimido; perda de interesse ou prazer; perda ou ganho significativo de peso; insônia ou hipersonia; agitação ou retardo psicomotor; fadiga ou perda de energia; sentimentos de inutilidade ou culpa excessiva; indecisão ou diminuição da capacidade para pensar ou se concentrar; e pensamentos recorrentes de morte ^9^. Além de serem fenômenos que inspiram cuidado na sociedade de uma maneira global, destaca-se que tanto o uso de mídias sociais como a prevalência de depressão vêm aumentando em populações de grupos etários mais jovens, como os estudantes universitários ^1^ ^,^ ^4^ ^,^ ^10^. Nessa população específica, observou-se uma prevalência de 33,6% com sintomas depressivos ^11^, além de estudos que encontraram associações entre depressão e o uso problemático de mídias sociais ^2^ ^,^ ^12^.

Embora não exista consenso na associação entre essas variáveis, a compreensão dessa associação depende de como o uso de mídias sociais é avaliado. De modo geral, existem três maneiras principais de realizar essa mensuração: (1) o uso em si (sim ou não), que é pouco discriminativo em populações que utilizam mídias sociais de forma muito prevalente; (2) o tempo de uso de mídias sociais; e (3) o uso problemático ^12^ ^,^ ^13^, quando apresenta características comportamentais e psicológicas compatíveis com o vício/dependência ^12^ ^,^ ^14^ ^,^ ^15^. Baseado nesse entendimento, foi observado que os sintomas depressivos apresentam fraca associação com o tempo e intensidade do uso de mídias sociais ^13^ e moderada associação com o uso problemático ^12^ ^,^ ^13^ ^,^ ^16^.

De modo similar, estudos apontam que o maior tempo de uso de mídias sociais está associado a uma pior qualidade do sono ^7^ ^,^ ^17^, a qual pode ser decorrente da dependência de mídias sociais ^18^. Nesse caso, é importante destacar a bidirecionalidade entre essas variáveis, visto que indivíduos com dificuldade para adormecer são mais propensos ao uso das mídias, podendo ocasionar dependência ^19^ ^,^ ^20^. Ainda nesse sentido, seguindo um caminho bidirecional, a literatura indica que indivíduos com uma pior qualidade do sono apresentam maior probabilidade de ocorrência de sintomas depressivos ^21^ ^,^ ^22^, assim como a depressão demonstrou ser um forte preditor de incidência e persistência de queixas do sono ^23^. No que se refere aos universitários, o hábito de se deitar tarde da noite, combinado com o início matutino das aulas, resulta em um fenômeno conhecido como “*jet lag social*” ^24^. Esse desalinhamento temporal se traduz em uma redução tanto na quantidade quanto na qualidade do sono desses indivíduos ^24^ ^,^ ^25^.

Considerando o aumento no uso de mídias sociais ^4^ ^,^ ^5^ ^,^ ^6^, e a crescente prevalência dos sintomas depressivos, sobretudo na população jovem ^10^, a associação entre essas variáveis ^11^ torna-se ainda mais preocupante. Nesse contexto, a identificação de problemas relacionados à saúde mental, como a percepção de dependência de mídias sociais ^13^ e a alteração na qualidade do sono ^26^ ^,^ ^27^ ^,^ ^28^, como possíveis mediadores é necessária e auxilia o entendimento dos efeitos do uso das redes sociais nos sintomas depressivos, além de embasar a adoção de estratégias de redução de danos ^13^ ^,^ ^29^. Assim, o objetivo deste estudo foi verificar o papel mediador da dependência de mídias sociais e da qualidade do sono na associação entre o tempo de uso de mídias sociais e sintomas depressivos em universitários.

## Metodologia

### Desenho e estudo de população

**Figura 1:**
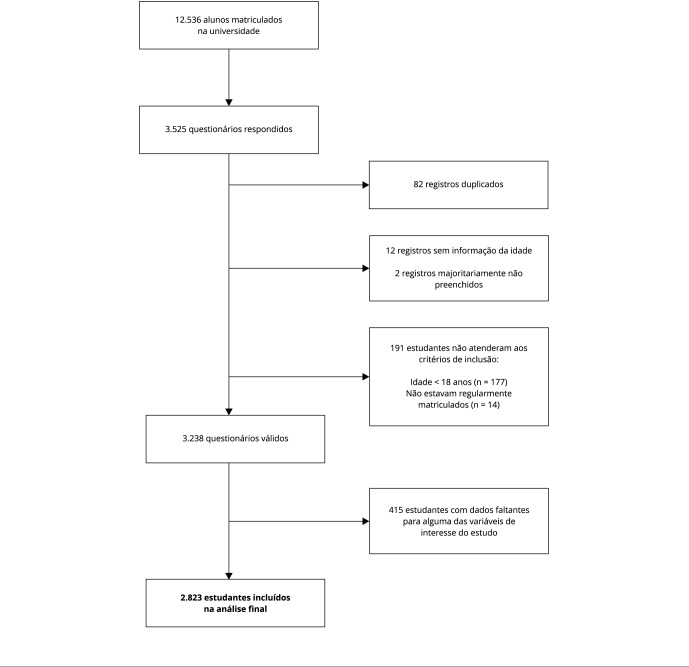
Fluxograma da população do estudo.

Trata-se de um estudo epidemiológico de delineamento transversal. Foram considerados elegíveis os estudantes da Universidade Estadual de Londrina (UEL), Paraná, com idade maior ou igual a 18 anos, regularmente matriculados na graduação no primeiro semestre de 2019. A coleta de dados foi realizada no período de abril a junho de 2019, por meio de um questionário anônimo no Google Forms (https://www.google.com/forms/), sendo precedida de uma ampla divulgação para incentivar a participação da população elegível ^30^. Embora o questionário não coletasse informações de identidade dos participantes, eles poderiam voluntariamente informar seu e-mail para receberem uma devolutiva individual das escalas empregadas na pesquisa. Com base nessa informação e na verificação de respostas com conteúdo repetido recebidas em um curto espaço de tempo, foram identificados registros duplicados. Ao final, foram excluídos registros duplicados (n = 82), que estavam majoritariamente não preenchidos (n = 2) e que não apresentavam informação da idade (n = 12). Dos 3.238 questionários válidos, 2.823 forneceram dados completos para as variáveis de interesse do estudo (Figura 1).

### Variáveis do estudo

A variável independente foi o tempo de uso de mídias sociais, obtido por meio da questão “Quantas horas por dia você geralmente gasta postando ou verificando mídias sociais? (WhatsApp, Facebook, Instagram, Twitter etc.)?”. Foram apresentadas as seguintes opções de resposta: não uso redes sociais; menos que 1 hora por dia; entre 1:00 e 1:59 hora por dia; entre 2:00 e 2:59 horas por dia; entre 3:00 e 4:59 horas por dia; entre 5:00 e 6:59 horas por dia; e 7 horas ou mais por dia.

Os sintomas depressivos, variável dependente, foram avaliados por meio do *Patient Health Questionnaire-9* (PHQ-9; Questionário de Saúde do Paciente), escala previamente adaptada e validada para a população brasileira ^31^, que permite o rastreamento de indivíduos com sintomas depressivos. O instrumento avalia a frequência com que os sintomas ocorreram durante as últimas duas semanas por nove questões com escalas que variam de 0 a 3, podendo a soma final alcançar de 0 a 27 pontos. Cada questão avalia a presença de cada sintoma clínico para o episódio de depressão maior, descritos no *Manual Diagnóstico e Estatístico de Transtornos Mentais* (DSM-5), como: “se sentiu cansado ou com pouca energia”. Para a análise descritiva, o ponto de corte para definir a presença de indicativo de depressão foi ≥ 9 pontos ^31^. Para a análise de mediação, foi utilizada a variável contínua.

Os potenciais mediadores foram a dependência de mídias sociais e a qualidade do sono. A dependência de mídias sociais foi obtida por meio de uma questão que aferiu a autopercepção da dependência “Qual a sua opinião sobre sua dependência de mídias sociais? (WhatsApp, Facebook, Instagram, Twitter etc.)?”. Foram apresentadas as seguintes opções de resposta: não uso redes sociais; não dependente; pouco dependente; indiferente; dependente; e muito dependente. Com base nas respostas dos participantes, eles foram classificados em três níveis de dependência: baixa (não dependente/pouco dependente); intermediária (indiferente); e alta (dependente/muito dependente). A qualidade do sono foi mensurada por meio do *Pittsburgh Sleep Quality Index* (PSQI; Índice de Qualdiade do Sono de Pittsburgh), instrumento composto por 19 questões categorizadas em sete componentes, os quais podem, cada um, ter pontuações de 0 a 3 pontos, resultando em um escore global de 0 a 21 pontos. Os sete componentes do instrumento contemplam: qualidade subjetiva do sono; latência para o sono (tempo que o indivíduo leva para efetivamente começar a dormir); duração do sono; eficiência habitual do sono (relação entre o tempo que se passa na cama com o tempo que se passa dormindo); transtornos do sono; uso de medicamentos para dormir; e disfunção diurna. Para a análise descritiva, a má qualidade do sono foi identificada pelo ponto de corte > 5 ^32^ ^,^ ^33^, ainda, um escore > 10 foi utilizado para identificar um possível distúrbio do sono ^34^. Para a análise de mediação, foi utilizada a variável contínua.

Variáveis com potencial efeito de confusão nas associações do estudo também foram avaliadas, tais como: sexo (feminino; masculino) ^13^; faixa etária em anos (18-20; 21-23; ≥ 24) ^13^; índice de massa corporal (IMC) ^35^ ^,^ ^36^, calculado a partir do peso autorreferido em quilogramas dividido pelo quadrado da altura em metros (kg/m^2^) e categorizado conforme classificação da Organização Mundial da Saúde (OMS) ^37^ (baixo peso; peso adequado; sobrepeso; obesidade); ingresso por cotas ^21^ ^,^ ^38^ (não; sim); período de estudo ^39^ (matutino; vespertino; noturno; integral; à distância); prática de atividade física ^40^, avaliada por meio da pergunta “Em uma semana típica, com qual frequência você pratica atividade física no seu tempo livre?” (não pratica; uma vez por semana; 2 a 3 vezes por semana; 4 ou mais vezes por semana); consumo de tabaco ^41^, avaliado por meio da pergunta “Durante os últimos três meses, com que frequência você fumou cigarro ou algum derivado do tabaco?” (nunca; 1 ou 2 vezes; mensalmente; semanalmente; diariamente ou quase todos os dias); consumo de substâncias ilícitas ^41^, avaliado por meio da pergunta “Durante os últimos três meses, com que frequência você utilizou substâncias ilícitas?” (nunca; 1 ou 2 vezes; mensalmente; semanalmente; diariamente ou quase todos os dias); e consumo de álcool ^41^, avaliado por meio da pergunta: “Durante os últimos três meses, com que frequência você utilizou bebidas alcoólicas?” (nunca; 1 ou 2 vezes; mensalmente; semanalmente; diariamente ou quase todos os dias).

### Análise estatística

Os dados foram analisados no programa SPSS, versão 19.0 para Windows (https://www.ibm.com/). Inicialmente, foi realizada a análise descritiva, com cálculo da média ± desvio padrão (DP), das variáveis contínuas e o número absoluto e frequência relativa (%) das variáveis categóricas. Foi feita uma análise de correlação de Spearman para verificar as associações bivariadas entre as variáveis do estudo, sendo considerado significativo p < 0,05. As variáveis com potencial efeito de confusão, ou seja, que apresentaram correlação com ao menos duas das variáveis de interesse, foram utilizadas como ajuste no modelo final da análise de mediação.

**Figura 2:**
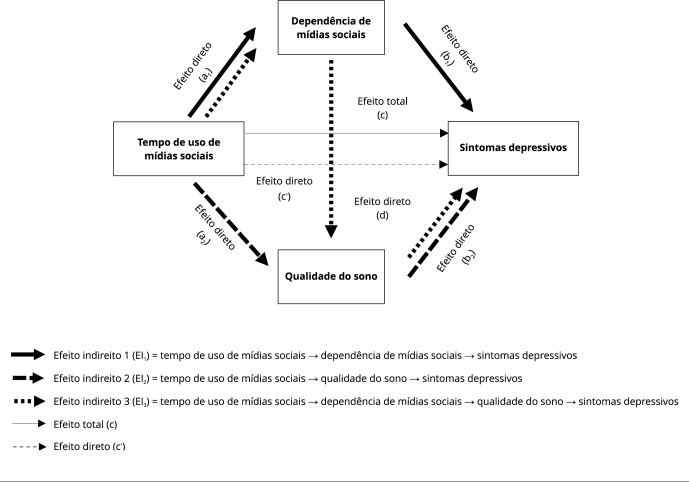
Modelo de mediação proposto para avaliar os efeitos total (c), diretos (a_1_, a_2_, b_1_, b_2_, d e c’) e indiretos (EI_1_, EI_2_ e EI_3_) entre o tempo de uso de mídias sociais e sintomas depressivos.

Foi realizada uma análise para verificar o possível efeito de mediação da dependência de mídias sociais e da qualidade do sono na associação entre o tempo de uso de mídias sociais e os sintomas depressivos. Para essa análise, foi utilizada a macro PROCESS, versão 4.1 (https://www.processmacro.org/index.html), para o programa SPSS. Utilizou-se o modelo 6, com dois mediadores, e 5 mil amostras de *bootstrap*, com intervalo de 95% de confiança (IC95%) ^42^. A dependência de mídias sociais foi considerada como primeiro mediador e a qualidade do sono como segundo. O modelo de mediação foi utilizado para avaliar os efeitos total (c) e direto (a_1_, a_2_, b_1_, b_2_, d e c’) que indicam o coeficiente de regressão não padronizado e a significância entre o tempo de uso de mídias sociais e os sintomas depressivos, sendo um valor de p < 0,05 considerado significativo. Ainda, esse modelo avaliou três efeitos indiretos (EI_1_, EI_2_ e EI_3_) que indicam a significância do efeito de mediação, os quais foram considerados significativos quando o IC95% não continha 0 ^42^ (Figura 2). Esta análise foi ajustada por sexo ^13^, idade ^13^, IMC ^35^ ^,^ ^36^, ingresso por cotas ^21^ ^,^ ^38^, período de estudo ^39^, prática de atividade física ^40^, e consumo de tabaco ^41^, de álcool ^41^ e de substâncias ilícitas ^41^.

Nota: para EI_1_ considerou-se o efeito da dependência de mídias sociais, para EI_2_ o efeito da qualidade do sono e para EI_3_ o efeito da dependência de mídias sociais e da qualidade do sono.

### Aspectos éticos

Todos os participantes foram devidamente informados quanto aos objetivos da pesquisa e só poderiam preencher o questionário após a leitura e aceitação do termo de consentimento livre e esclarecido (TCLE). O projeto foi aprovado pelo Comitê de Ética em Pesquisa da UEL (certificado de apresentação para apreciação ética nº 04456818.0.0000.5231).

## Resultados

Dos 2.823 estudantes incluídos no estudo (Figura 1), a idade média foi 22,0 ± 4,5 anos, sendo 68,7% do sexo feminino. A maioria (59,2%) apresentou peso adequado e, em relação às características acadêmicas, 63,7% não ingressou na universidade por cotas e 44,8% dos indivíduos frequentavam cursos de período integral. No que se refere aos hábitos de vida e ao consumo de substâncias nos últimos três meses, 74,4% relataram não utilizar cigarro ou derivados do tabaco, 73,3% disseram não consumir substâncias ilícitas e 31,4% referiram consumir álcool semanalmente. Além disso, 52,7% referiram não praticar atividade física. Em relação ao uso de mídias sociais, 53,1% referiram alta dependência e 26,1% afirmaram gastar entre 2:00 e 2:59 horas por dia com mídias sociais (Tabela 1).

**Tabela 1:** Caracterização da amostra do estudo (n = 2.823).

Variáveis	n (%)
Sexo	
Feminino	1.939 (68,7)
Masculino	884 (31,3)
Faixa etária (anos)	
18-20	1.268 (44,9)
21-23	971 (34,4)
≥ 24	584 (20,7)
IMC (kg/m^2^)	
Baixo peso (< 18,5)	260 (9,2)
Peso adequado (18,5 a < 25,0)	1.671 (59,2)
Sobrepeso (25,0 a < 30,0)	612 (21,7)
Obesidade (≥ 30,0)	280 (9,9)
Ingresso por cotas	
Não	1.799 (63,7)
Sim	1.024 (36,3)
Período de estudo	
Matutino	627 (22,2)
Vespertino	135 (4,8)
Noturno	780 (27,6)
Integral	1.265 (44,8)
À distância	16 (0,6)
Prática de atividade física no tempo livre	
Não pratica	1.489 (52,7)
1 vez por semana	441 (15,6)
2 a 3 vezes por semana	564 (20,0)
4 ou mais vezes por semana	329 (11,7)
Consumo de tabaco	
Nunca	2.100 (74,4)
1 ou 2 vezes	322 (11,4)
Mensalmente	106 (3,7)
Semanalmente	126 (4,5)
Diariamente ou quase todos os dias	169 (6,0)
Consumo de álcool	
Nunca	598 (21,2)
1 ou 2 vezes	717 (25,4)
Mensalmente	552 (19,6)
Semanalmente	887 (31,4)
Diariamente ou quase todos os dias	69 (2,4)
Consumo de substâncias ilícitas	
Nunca	2.068 (73,3)
1 ou 2 vezes	457 (16,1)
Mensalmente	118 (4,2)
Semanalmente	113 (4,0)
Diariamente ou quase todos os dias	67 (2,4)
Tempo de uso de mídias sociais por dia	
Não uso mídias sociais	21 (0,7)
Menos que 1 hora	305 (10,8)
Entre 1:00 e 1:59 horas	675 (23,9)
Entre 2:00 e 2:59 horas	738 (26,2)
Entre 3:00 e 4:59 horas	663 (23,5)
Entre 5:00 e 6:59 horas	243 (8,6)
Mais que 7 horas	178 (6,3)
Dependência de mídias sociais	
Baixa	887 (31,4)
Intermediária	438 (15,5)
Alta	1.498 (53,1)
Qualidade do sono *	
Boa	680 (24,1)
Ruim	2.143 (75,9)
Média ± DP	7,9 ± 3,1
Indicativo de depressão **	
Não	746 (26,4)
Sim	2.077 (73,6)
Média ± DP	13,7 ± 6,7

DP: desvio padrão; IMC: índice de massa corporal.

* Verificada pelo *Pittsburgh Sleep Quality Index* (PSQI): ponto de corte > 5 (escore 0 a 21);

** Verificado pelo *Patient Health Questionnaire-9* (PHQ-9): ponto de corte ≥ 9 (escore 0 a 27).

Observou-se, ainda, que 73,6% dos indivíduos apresentaram indicativo de depressão (Tabela 1), sendo que 42,9% referiram se sentir cansados ou com pouca energia quase todos os dias nas últimas duas semanas (Tabela 2). Com relação ao sono, 75,9% apresentaram má qualidade do sono (Tabela 1) e, entre eles, 25% obtiveram pontuação compatível com um possível distúrbio do sono. Na análise descritiva dos componentes do PSQI, observou-se que 57,9% dos indivíduos referiram duração do sono entre 6 e 7 horas (Tabela 3).

**Tabela 2:** Frequência dos sintomas avaliados pelo *Patient Health Questionnaire-9* (PHQ-9), referentes às últimas duas semanas (n = 2.823).

Sintomas	Nenhum dia	Menos de uma semana	Uma semana ou mais	Quase todos os dias
	n (%)	n (%)	n (%)	n (%)
Teve pouco interesse ou pouco prazer em fazer as coisas	210 (7,4)	969 (34,3)	787 (27,9)	857 (30,4)
Se sentiu para baixo, deprimido ou sem perspectiva	371 (13,1)	969 (34,3)	822 (29,2)	661 (23,4)
Teve dificuldade para pegar no sono ou permanecer dormindo ou dormiu mais do que de costume	576 (20,4)	824 (29,2)	696 (24,7)	727 (25,7)
Se sentiu cansado ou com pouca energia	103 (3,6)	646 (22,9)	863 (30,6)	1.211 (42,9)
Teve falta de apetite ou comeu demais	544 (19,3)	665 (23,6)	734 (26,0)	880 (31,1)
Se sentiu mal consigo mesmo ou achou que é um fracasso ou que decepcionou sua família ou a você mesmo	621 (22,0)	727 (25,8)	663 (23,4)	812 (28,8)
Teve dificuldade para se concentrar nas coisas (como ler o jornal ou ver televisão)	469 (16,6)	793 (28,1)	774 (27,4)	787 (27,9)
Teve lentidão para se movimentar ou falar (a ponto das outras pessoas perceberem), ou ao contrário, esteve tão agitado que você ficava andando de um lado para o outro mais do que de costume	1.266 (44,8)	688 (24,4)	477 (16,9)	392 (13,9)
Pensou em se ferir de alguma maneira ou que seria melhor estar morto	1.898 (67,2)	430 (15,2)	273 (9,7)	222 (7,9)

**Tabela 3:** Frequência de respostas das categorias referentes a cada um dos componentes do *Pittsburgh Sleep Quality Index* (PSQI) (n = 2.823).

Componente	n (%)
Qualidade subjetiva do sono	
Muito boa	178 (6,3)
Boa	1.372 (48,6)
Ruim	1.039 (36,8)
Muito ruim	234 (8,3)
Latência para o sono	
0	511 (18,1)
1	1.009 (35,7)
2	840 (29,8)
3	463 (16,4)
Duração do sono (horas)	
≥ 7	552 (19,6)
≥ 6 a < 7	1.635 (57,9)
≥ 5 a < 6	480 (17,0)
< 5	156 (5,5)
Eficiência habitual do sono (%)	
≥ 85	2.291 (81,2)
75-84	372 (13,2)
65-74	111 (3,9)
< 65	49 (1,7)
Transtornos do sono	
0	97 (3,4)
1	1.754 (62,2)
2	887 (31,4)
3	85 (3,0)
Uso de medicamentos para dormir	
Nenhuma vez no último mês	2.262 (80,2)
Menos de 1 vez por semana	232 (8,2)
1 ou 2 vezes por semana	130 (4,6)
3 ou mais vezes por semana	199 (7,0)
Disfunção diurna	
0	107 (3,8)
1	850 (30,1)
2	1.178 (41,7)
3	688 (24,4)

**Figura 3:**
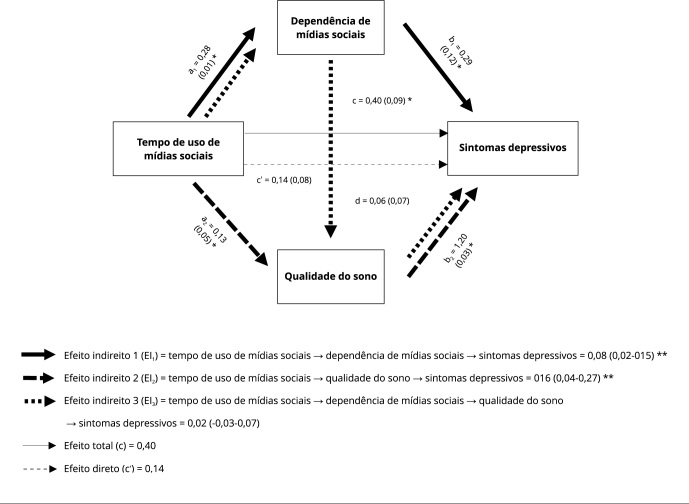
Análise de mediação da associação entre tempo de uso de mídias sociais e sintomas depressivos, utilizando como mediadores dependência de mídias sociais e qualidade do sono.

A dependência de mídias sociais, a qualidade do sono, o tempo de uso de mídias sociais e os sintomas depressivos apresentaram correlações significativas entre si. Além disso, essas variáveis apresentaram correlação com muitas das variáveis com potencial efeito de confusão, conforme apresentado na Tabela 4. Com relação à análise de mediação (Figura 3), evidenciou-se que há uma associação entre o tempo de uso de mídias sociais e os sintomas depressivos. No entanto, observou-se que o efeito direto (c’ = 35%) entre essas variáveis não foi significativo, sendo essa associação mediada pela dependência de mídias sociais e pela qualidade do sono. Os efeitos indiretos 1 (EI_1_ = 0,08; IC95%: 0,02-0,15) e 2 (EI_2_ = 0,16; IC95%: 0,04-0,27) foram as vias estatisticamente significativas para essa mediação. Dessa forma, é possível identificar que 20% e 40% do efeito do tempo de uso de mídias sociais nos sintomas depressivos foram mediados, respectivamente, pela dependência de mídias sociais e pela má qualidade do sono.

**Tabela 4:** Correlação entre variáveis com potencial efeito de confusão, qualidade do sono, dependência de mídias sociais, tempo de uso de mídias sociais e sintomas depressivos.

Variáveis	1	2	3	4	5	6	7	8	9	10	11	12	13
1. Sexo	1,00												
2. Idade	-0,06 *	1,00											
3. IMC	-0,10 **	0,14 **	1,00										
4. Ingresso por cotas	0,01	-0,05 ***	0,04 ***	1,00									
5. Período de estudo	-0,03	0,04 ***	-0,07 **	0,01	1,00								
6. Prática de atividade física no tempo livre	-0,17 **	0,002	-0,06 ***	-0,11 **	-0,04 ***	1,00							
7. Consumo de tabaco	-0,12 **	0,04	0,04	-0,09 **	-0,07 **	0,04 ***	1,00						
8. Consumo de álcool	-0,09 **	0,04	-0,01	-0,09 **	-0,01	0,09 **	0,45 **	1,00					
9. Consumo de substâncias ilícitas	-0,11 **	0,02	-0,01	-0,05 ***	-0,05 ***	0,07 **	0,56 **	0,45 **	1,00				
10. Tempo de uso de mídias sociais	0,16 **	-0,13 **	0,02	-0,02	-0,08 **	-0,04	0,08 **	0,14 **	0,12 **	1,00			
11. Dependência de mídias sociais	0,13 **	-0,09 **	-0,06 ***	-0,05 ***	-0,04 ***	0,00	0,05 ***	0,13 **	0,10 **	0,46 **	1,00		
12. Qualidade do sono	0,14 **	0,08 **	0,08 **	0,05 ***	0,01	-0,14 **	0,12 **	0,06 *	0,10 **	0,10 **	0,07 **	1,00	
13. Sintomas depressivos	0,21 **	-0,01	0,07 **	0,05 ***	-0,02	-0,19 **	0,14 **	0,08 **	0,13 **	0,14 **	0,11 **	0,61 **	1,00

IMC: índice de massa corporal.

Nota: sexo: 0 = masculino, 1 = feminino; idade: 0 = 18-20 anos, 1 = 21-23 anos, 2 = ≥ 24 anos; IMC: 0 = peso adequado, 1 = baixo peso, 2 = sobrepeso, 3 = obesidade; ingresso por cotas: 0 = não, 1 = sim; período de estudo: 0 = matutino, 1 = vespertino, 2 = noturno, 3 = integral, 4 = à distância; prática de atividade física no tempo livre: 0 = não pratica, 1 = 1 vez por semana, 2 = 2 a 3 vezes por semana, 3 = 4 ou mais vezes por semana; para consumo de tabaco, de álcool e de substâncias ilícitas: 0 = nunca, 1 = 1 ou 2 vezes, 3 = mensalmente, 4 = semanalmente, 5 = diariamente ou quase todos os dias; dependência de mídias sociais: 0 = dependência baixa, 1 = dependência intermediária, 2 = dependência alta; tempo de uso de mídias sociais por dia: 0 = não uso mídias sociais, 1 = menos que 1:00 hora, 2 = entre 1:00 e 1:59 hora, 3 = entre 2:00 e 2:59 horas, 4 = entre 3:00 e 4:59 horas, 5 = entre 5:00 e 6:59 horas, 6 = mais que 7:00 horas. Para qualidade do sono e sintomas depressivos, foram utilizadas variáveis contínuas, sendo que maiores escores indicam maior frequência.

* p < 0,01;

** p < 0,001;

*** p < 0,05.

Nota: o modelo foi ajustado por sexo, idade, índice de massa corporal, ingresso por cotas, período de estudo, prática de atividade física, e consumo de tabaco, de álcool e de substâncias ilícitas. Os caminhos a_1_, a_2_, b_1_, b_2_, d, c e c’ são expressos como o coeficiente de regressão não padronizado (erro padrão). EI_1_, EI_2_ e EI_3_ são expressos como coeficientes de regressão não padronizados (intervalos de 95% de confiança). Para tempo de uso de mídias sociais por dia: 0 = não uso mídias sociais, 1 = menos que 1:00 hora, 2 = entre 1:00 e 1:59 hora, 3 = entre 2:00 e 2:59 horas, 4 = entre 3:00 e 4:59 horas, 5 = entre 5:00 e 6:59 horas, 6 = mais que 7:00 horas; dependência de mídias sociais: 0 = dependência baixa, 1 = dependência intermediária, 2 = dependência alta. Para qualidade do sono e sintomas depressivos, foram utilizadas variáveis contínuas, sendo que maiores escores indicam maior frequência. * p < 0,05; ** Efeito estatisticamente significativo.

## Discussão

Os resultados deste estudo evidenciaram uma associação entre o tempo de uso de mídias sociais e os sintomas depressivos, a qual foi mediada pela dependência de mídias sociais e pela qualidade do sono, independentemente de fatores de confusão. Estudos apontam elevados índices de depressão decorrentes do aumento do uso das mídias sociais ^2^ ^,^ ^8^, fato também observado nesta pesquisa, a partir dos efeitos total e direto do tempo de uso de mídias sociais nos sintomas depressivos. No entanto, observa-se que é um efeito complexo, influenciado por diversos fatores, que, por sua vez, podem se associar aos sintomas depressivos ^18^ ^,^ ^28^ ^,^ ^41^.

Nesse sentido, em razão da mediação identificada (caminhos EI_1_ e EI_2_), torna-se relevante o entendimento desses fatores e dos possíveis mecanismos intermediários existentes na associação entre o tempo de uso de mídias sociais e os sintomas depressivos. Assim, o EI_1_, mediado pela dependência de mídias sociais, pode indicar que indivíduos que permanecem mais tempo conectados tendem a ser mais dependentes ^18^ ^,^ ^26^, com tendência a apresentar problemas emocionais e falta de prazer nas atividades diárias ^28^ ^,^ ^43^. Além disso, a dependência de mídias sociais está associada com o “medo de perder algo”, caracterizado pelo desejo de estar continuamente inteirado das novidades ^44^, e com a presença e a gravidade dos sintomas depressivos ^45^. Ainda nesse contexto, é importante ressaltar que há uma tendência de os indivíduos dependentes sobreporem múltiplos vícios ^46^. Desse modo, o consumo de substâncias como tabaco, álcool e drogas ilícitas podem predispor à dependência de mídias sociais ^47^, assim como a dependência de mídias sociais pode desencadear o maior uso dessas substâncias ^41^ ^,^ ^48^ ^,^ ^49^. Logo, essa bidirecionalidade e a possível sobreposição desses comportamentos podem contribuir ainda mais para o aparecimento de sintomas depressivos ^41^.

Por sua vez, o EI_2_ aponta que a qualidade do sono também atua como mediadora na associação entre o tempo de uso de mídias sociais e os sintomas depressivos. Indivíduos que permanecem mais tempo conectados tendem a apresentar distúrbios do sono ^7^ ^,^ ^17^ ^,^ ^50^. Nesse contexto, recente revisão sistemática evidenciou que o uso de mídias digitais foi associado a menor duração e pior qualidade do sono ^51^. Ainda, o uso do celular, principalmente no período noturno, está associado a menor qualidade e tempo de duração do sono, com mais despertares noturnos, maior latência do sono e sonolência diurna, que podem favorecer o desenvolvimento de sintomas depressivos ^7^. Os efeitos do tempo de uso de mídias sociais sobre a qualidade do sono são atribuídos à exposição noturna à luz do *smartphone*, que pode afetar o sistema de temporização do ciclo sono-vigília e a liberação da melatonina, resultando em alterações psicológicas e elevados níveis de depressão ^7^ ^,^ ^52^, além da associação entre o maior tempo de uso de mídias sociais e um menor tempo disponível para dormir ^53^ ^,^ ^54^. Ainda, destaca-se que os universitários correspondem a uma faixa etária com tendência à vespertinidade ^55^; assim, o desafio temporal que compreende o deitar-se tarde da noite e levantar-se cedo pela manhã para o início das aulas pode contribuir para a ocorrência de prejuízos nos padrões de sono ^56^. Por fim, embora haja evidências de associação entre dependência do celular e distúrbios do sono ^18^, neste estudo a percepção de dependência não interferiu na qualidade do sono, motivo pelo qual o caminho EI_3_, que passa por ambos os mediadores concomitantemente, não se mostrou significativo.

Além dos fatores estudados, ressalta-se a influência de outros mecanismos na associação entre o tempo de uso de mídias sociais e os sintomas depressivos, relacionados a alterações no comportamento devido ao uso de mídias sociais ou a alterações psicológicas advindas da exposição ^40^ ^,^ ^57^ ^,^ ^58^. Inicialmente, o maior tempo de uso de mídias sociais pode significar uma diminuição no tempo dedicado aos estudos, haja vista que o design das redes é projetado para ser atrativo e manter os usuários o máximo de tempo conectados ^59^, o que pode tornar esses estudantes mais suscetíveis a problemas acadêmicos e transtornos mentais ^60^. Ainda, indivíduos que passam mais tempo conectados se envolvem menos em outras atividades que proporcionam bem-estar, como atividades físicas ^40^ ^,^ ^61^ e interações sociais presenciais ^60^ ^,^ ^62^, o que pode contribuir para o aumento de depressão ^40^ ^,^ ^60^ ^,^ ^61^ ^,^ ^62^.

As alterações psicológicas advindas da exposição às mídias sociais podem ser justificadas pelo consumo de determinados conteúdos, como aqueles que retratam realidades tidas como ideais (de beleza, moradia, viagens, consumo, entre outras) pelos padrões sociais hegemônicos, os quais provocam comparações que podem gerar, por sua vez, um sentimento de inferioridade, com consequentes efeitos negativos sobre a saúde mental dos indivíduos ^58^ ^,^ ^63^. Ademais, a exposição às mídias pode aumentar os níveis de insatisfação corporal, gerando impacto negativo na autoestima e no estado de humor ^64^.

Ressalta-se que, para uma correta compreensão das associações identificadas, é importante avaliar os possíveis efeitos das variáveis com potencial efeito de confusão. Nesse sentido, parece haver uma intrincada rede de associações entre essas variáveis, na qual a inatividade física e o maior IMC parecem se associar de forma bidirecional ^65^, sendo mais observados em indivíduos que passam mais tempo conectados ^36^ ^,^ ^40^ ^,^ ^66^ e apresentam sintomas depressivos ^40^ ^,^ ^66^, além de estar associada a uma pior qualidade do sono ^66^ ^,^ ^67^. Ainda, destaca-se que o consumo de substâncias, como tabaco, álcool e substâncias ilícitas, apresenta-se frequentemente associado, em uma relação bidirecional, com o uso de mídias sociais ^41^, a depressão ^68^ e a pior qualidade do sono ^69^. Nota-se que muitas dessas associações puderam ser evidenciadas por meio de correlações nos resultados obtidos, por exemplo: entre IMC e inatividade física, menor dependência de mídias sociais, pior qualidade do sono e mais sintomas depressivos. Assim, embora estas análises tenham sido ajustadas por uma série de potenciais fatores de confusão, diante da complexa e multidirecional relação existente entre as variáveis principais e as covariáveis analisadas, é prudente considerar a possível persistência de confusão residual.

Este estudo apresenta algumas limitações que devem ser consideradas. Inicialmente, o desenho transversal não permite o estabelecimento de temporalidade, o que pode ocasionar viés de causalidade reversa. Ainda, ressalta-se o fato de serem variáveis autorreferidas, sendo o tempo de uso de mídias sociais e a dependência de mídias sociais estimados de forma subjetiva, podendo haver viés de informação. Também se considera como limitação o fato de não ter avaliado o tempo de uso que os participantes faziam em cada mídia social, a finalidade (estudos, lazer ou trabalho) e o conteúdo acessado, bem como variáveis relacionadas à percepção psicológica dos participantes, que poderiam aumentar o poder explicativo das análises. Contudo, destaca-se como fortalezas deste estudo o elevado número de participantes, a realização de análises ajustadas por possíveis fatores de confusão e a coesão entre os resultados encontrados e os mecanismos que explicam a associação entre o uso de mídias sociais e sintomas depressivos.

Para a confirmação dos resultados apresentados e aprofundamento do tema, considera-se relevante o desenvolvimento de estudos prospectivos, para explorar a causalidade da relação entre o tempo de uso de mídias sociais e os sintomas depressivos, bem como confirmar o possível papel da dependência de mídias sociais e da qualidade do sono nessa relação em jovens adultos. Além disso, julga-se importante que mais estudos sejam realizados levando em consideração o uso de cada mídia social separadamente, a finalidade e os conteúdos acessados, além de variáveis que possam indicar possíveis mecanismos mediadores por meio da alteração da percepção psicológica dos participantes.

## Conclusão

Os resultados deste estudo indicam que a associação entre o tempo de uso de mídias sociais e os sintomas depressivos em universitários, independentemente de variáveis de confusão, é mediada pela dependência de mídias sociais e pela qualidade do sono. Os achados permitiram ampliar o conhecimento acerca de diferentes mecanismos que se influenciam mutuamente para explicar essa associação. Assim, estratégias devem ser adotadas para a redução dos danos, como campanhas para alertar sobre os efeitos nocivos do uso de mídias sociais na saúde mental, além da conscientização para reduzir o tempo de uso e organizar esse tempo para a realização de outras atividades que promovam bem-estar e incentivem uma melhora na qualidade do sono dos estudantes.
